# Direct Anterior Approach for One-Stage Bilateral Total Hip Arthroplasty in an ASA 3 Wheelchair-Dependent Woman

**DOI:** 10.1155/2019/5183578

**Published:** 2019-10-13

**Authors:** B. (Britt) Barvelink, J. T. (Arjan) Hooghof, R. B. G. (Roy) Brokelman

**Affiliations:** Deventer Ziekenhuis, Netherlands

## Abstract

This case report involves a 79-year-old wheelchair-dependent woman with bilateral destructive coxarthrosis, requiring total hip arthroplasty (THA). Mobilization and transfers were unbearable due to the bilateral involvement of her hips. Performing unilateral THA would not be sufficient due to the coexisting pain from the contralateral side. Therefore, the decision was made to perform bilateral THA in one stage using the direct anterior approach (DAA). One-stage bilateral THA (1-SBTHA) using the DAA in ASA 3 patients is not previously described in the literature. The procedure was completed as planned, without any major perioperative complications. Eight weeks postoperatively, the patient was able to mobilize unaccompanied using a walker. She regained her mobility and independence. This outcome suggests that 1-SBTHA using DAA can be considered for disabling coxarthrosis in carefully selected ASA 3 patients. DAA is the superior approach for 1-SBTHA, due to decreased muscle damage leading to early mobilization with improved gait. Another benefit of DAA is that both hips can be draped simultaneously without repositioning the patient during the procedure.

## 1. Introduction

The question of whether bilateral coxarthrosis should be treated in a one- or two-stage procedure has been discussed since the 70s [1]. Recent literature comparing one-stage versus two-stage procedures in terms of complication and mortality rates in large study populations reported similar results [2–6]. Some studies even reported fewer postoperative systemic complications (e.g., deep venous thrombosis and cardiovascular and pulmonary complications) and better functional outcome (walking capacity) in the one-stage procedure [5, 6]. The overall complication rate in hip surgery is greatly influenced by the patient's preoperative health status, which is generally quantified using the American Society of Anesthesiology score (ASA). A systematic review of Haverkamp et al. [3] reported that one-stage bilateral total hip arthroplasty (1-SBTHA) is not associated with more complications in ASA 1 and 2 patients. Weinstein et al. [7] reported no increased mortality risk or major complications in patients aged 75 years or older, compared to a younger cohort. However, literature about 1-SBTHA performed in ASA 3 patients is scarce. The possible benefits of this procedure are shorter hospital stay and increased mobility and therefore faster recovery. Possible disadvantages are increased blood loss and increased need for analgesics.

## 2. Case Presentation

A 79-year-old female presented in our outpatient department with severe and disabling bilateral hip pain, which could no longer be appropriately managed with extensive analgesics. Her medical history showed (among others) chronic renal insufficiency resulting in anemia (Hb 5.1), insulin-dependent diabetes mellitus, and obesity (BMI 37). She was therefore classified as ASA 3. She had been wheelchair dependent for the past two years, with progressive night and rest pain. Transfers (to wheelchair and bed/chair) were nearly impossible due to excessive pain.

**Figure 1:**
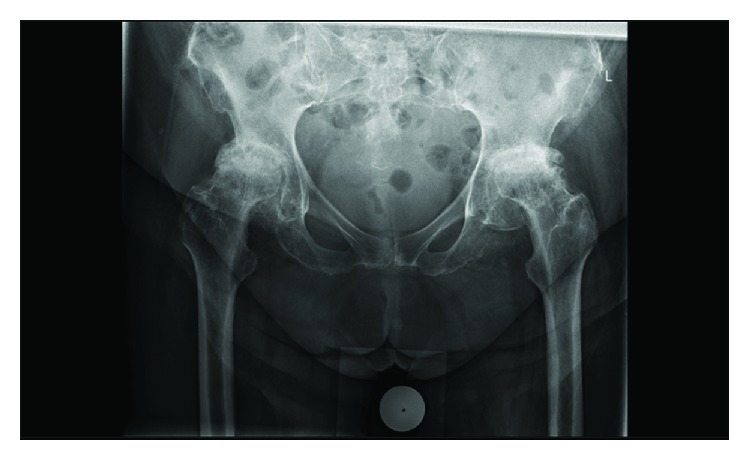
Preoperative anteroposterior X-ray of the pelvis.

On clinical examination, the patient was unable to walk and could stand for only a few seconds. Both hips showed severely limited range of motion with bilateral fixed flexion deformities of 30 degrees. Rotations were restricted and accompanied by severe groin pain. Neurovascular status was normal. Radiographs of the pelvis showed severe bilateral coxarthrosis with destruction of both femoral heads (Figure 1).

Due to the bilateral involvement of the hips, improvement of gait after unilateral THA was not expected. Therefore, we obtained consent from the patient and performed a one-stage bilateral THA (1-SBTHA) using the direct anterior approach (DAA). The DAA has a major advantage over other techniques (e.g., the posterolateral hip approach) in 1-SBTHA because both hips can be draped concurrently, and there is no need to reposition the patient. In case of significant blood loss (>500 cc) during the first THA, the contralateral THA could be postponed.

**Figure 2:**
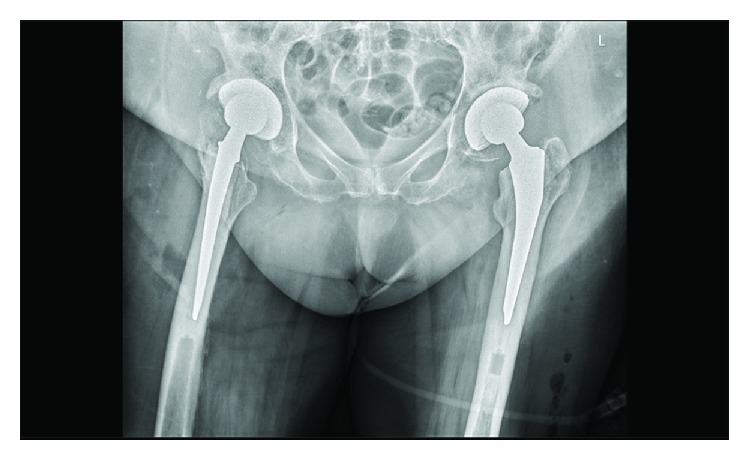
Postoperative anteroposterior X-ray of the pelvis.

The patient underwent surgery in the supine position under spinal anesthesia. Both hips were replaced using a 54 mm and 56 mm Allofit cup (Zimmer Biomet) on the right and left side, respectively. A 32 mm cross-linked polyethylene acetabular liner and 32 mm ceramic head were used as bearings on both sides. A cemented Muller straight stem 7.5 was placed in both femurs (Zimmer Biomet). Perioperatively, a minor iatrogenic fracture of the right greater trochanter occurred, leading to exorotation of the right hip postoperatively (Figure 2). The operating time was 132 min in total, with an estimated blood loss of 650 ml. A cell saver was used during the procedure. Postoperative Hb was 5.1 mmol/l, and renal function remained stable. Two units of red blood cell concentrate were administered on the first postoperative day resulting in Hb of 6.2 mmol/l. Rehabilitation started several hours after surgery by sitting in a chair. By day three, the patient was able to walk under guidance of a physiotherapist using a walker. The iatrogenic fracture of the right greater trochanter was of no influence on the rehabilitation process. She was discharged to a nursing home after 5 days where she rehabilitated without any further complications.

**Figure 3:**
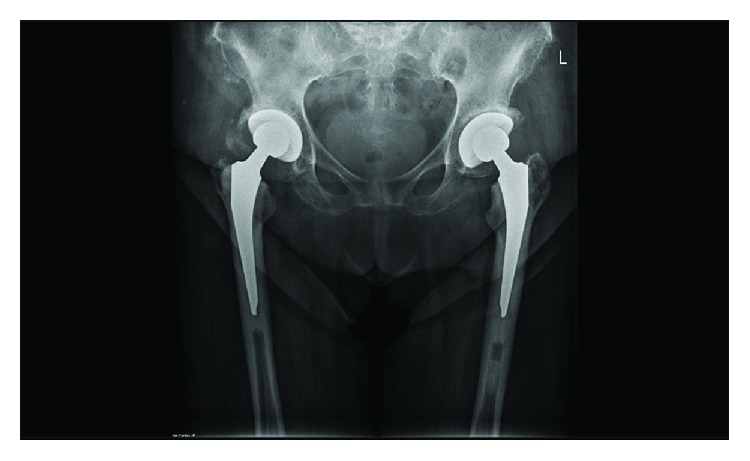
8 weeks of follow-up anteroposterior X-ray of the pelvis.

During the 8 weeks of postoperative period, the patient's rehabilitation progressed without concern. On a follow-up examination, hip rotation was painless bilaterally. The X-ray showed well-positioned bilateral hybrid total hip prostheses (Figure 3). The right hip showed a displaced greater trochanter fragment, as previously described. The Harris Hip Score improved from 10 preoperatively to 66 postoperatively and from 15 to 70 on the right and left side, respectively [8]. The Oxford Hip Score improved for both hips from 4 preoperatively to 30 postoperatively [9]. Although the patient was able to ambulate indoors with a walker, she was unable to continue beyond 10 minutes, due to deconditioning from her previous medical conditions. Despite this, the patient was quite satisfied with the outcomes, as her pain was significantly reduced, which allowed her to regain some of her independence.

## 3. Discussion

In the Netherlands, there will be a demographic shift with a fivefold increase of people over 75 years of age in the next 30 years [10]. The need for unilateral and bilateral THAs will be doubled or even tripled by that time. In the case of bilateral hip osteoarthrosis, pain control and functional outcome will be suboptimal until arthroplasty is performed bilaterally. The advantages and disadvantages of one-stage versus two-stage bilateral THA have been discussed for decades [11]. Single anesthetic administration, shorter hospital stay, faster rehabilitation, and lower procedure costs support 1-SBTHA. However, it remains controversial due to the safety concerns regarding the anesthetic risks and increased blood loss associated with 1-SBTHA [12]. A large study using prospectively collected data by Aghayev et al. [5] reported fewer postoperative local and systemic complications and a superior outcome in terms of postoperative walking capacity in the 1-SBTHA procedure versus the two-stage THA procedure (2-SBTHA). Shao et al. [6] published the same findings, with a significantly lower risk of major systemic postoperative complications and no difference in mortality rates. Weinstein et al. [7] reported that patients over 75 years of age, who underwent 1-SBTHA, have no increased inpatient mortality risk compared to a younger cohort and have excellent functional outcome after 2.5 years of follow-up. However, other articles have shown that bilateral THA is associated with higher risk of systemic complications, especially pulmonary complications [13, 14]. Due to this controversy, preoperative screening and careful patient selection are of utmost importance. As mentioned earlier, the literature regarding ASA 3 patients is scarce. The nationwide Swedish study performed by Garland et al. observed early postoperative mortality after 1-SBTHA and staged THA in 42,238 patients. Using subcohort analysis, ASA 3-5 was found to be a risk factor for an increased 90-day mortality risk compared to ASA 1 [2]. However, no comparison was made between ASA 2 and ASA 3-5 and the incidence of ASA 3-5 in their population was low (13.2%). To our knowledge, no research has been published concerning the outcome of 1-SBTHA performed in ASA 3 patients using the DAA. The DAA is gaining popularity, which as seen in the Dutch LROI (platform register orthopedic implants) [15]. The DAA claims to cause less muscle damage leading to quicker recovery with less pain, early mobilization, and improved gait [16]. Also, the DAA is associated with significantly less blood loss and lower blood transfusion rates compared to the direct lateral approach [17]. Another advantage of the DAA is that both hips can be draped simultaneously and surgery can be performed without repositioning the patient which is a significant advantage in 1-SBTHA. In this case, the patient regained mobility and independence within 8 weeks. It is likely that her mobility will increase further on in the next few months. This case suggests that 1-SBTHA using DAA can be a safe and effective treatment in destructive bilateral coxarthrosis in ASA 3 patients.

### 3.1. Learning Points

- One-stage bilateral DAA total hip arthroplasty can be a safe and effective treatment in ASA 3 patients with destructive coxarthrosis, leading to quicker mobility and independence
- This case highlights the advantages of 1-SBTHA using the direct anterior approach in fragile ASA 3 patients. Vital anesthetic time is conserved by not repositioning and redraping the patient
- The incidence of (bilateral) coxarthrosis will increase because of the demographic shift in age, making one-stage bilateral total hip arthroplasty a hot topic in orthopedic surgery
